# OptEF-BCI: An Optimization-Based Hybrid EEG and fNIRS–Brain Computer Interface

**DOI:** 10.3390/bioengineering10050608

**Published:** 2023-05-18

**Authors:** Muhammad Umair Ali, Kwang Su Kim, Karam Dad Kallu, Amad Zafar, Seung Won Lee

**Affiliations:** 1 Department of Intelligent Mechatronics Engineering, Sejong University, Seoul 05006, Republic of Korea; umair@sejong.ac.kr; 2 Department of Scientific Computing, Pukyong National University, Busan 48513, Republic of Korea; kwangsukim@pknu.ac.kr; 3 Interdisciplinary Biology Laboratory (iBLab), Division of Biological Science, Graduate School of Science, Nagoya University, Nagoya 464-8602, Japan; 4 Department of Robotics & Artificial Intelligence (R&AI), School of Mechanical and Manufacturing Engineering (SMME), National University of Sciences and Technology (NUST) H-12, Islamabad 44000, Pakistan; karamdad.kallu@smme.nust.edu.pk; 5 Department of Precision Medicine, School of Medicine, Sungkyunkwan University, Suwon 16419, Republic of Korea

**Keywords:** optimal feature selection, hybrid BCI, binary enhanced whale optimization algorithm, fNIRS, EEG

## Abstract

Multimodal data fusion (electroencephalography (EEG) and functional near-infrared spectroscopy (fNIRS)) has been developed as an important neuroimaging research field in order to circumvent the inherent limitations of individual modalities by combining complementary information from other modalities. This study employed an optimization-based feature selection algorithm to systematically investigate the complementary nature of multimodal fused features. After preprocessing the acquired data of both modalities (i.e., EEG and fNIRS), the temporal statistical features were computed separately with a 10 s interval for each modality. The computed features were fused to create a training vector. A wrapper-based binary enhanced whale optimization algorithm (E-WOA) was used to select the optimal/efficient fused feature subset using the support-vector-machine-based cost function. An online dataset of 29 healthy individuals was used to evaluate the performance of the proposed methodology. The findings suggest that the proposed approach enhances the classification performance by evaluating the degree of complementarity between characteristics and selecting the most efficient fused subset. The binary E-WOA feature selection approach showed a high classification rate (94.22 ± 5.39%). The classification performance exhibited a 3.85% increase compared with the conventional whale optimization algorithm. The proposed hybrid classification framework outperformed both the individual modalities and traditional feature selection classification (*p* < 0.01). These findings indicate the potential efficacy of the proposed framework for several neuroclinical applications.

## 1. Introduction

Brain–computer interfaces (BCIs) enable interaction with the environment using brain signals, without the involvement of muscles or the peripheral nervous system [1,2,3]. BCI have flourished and developed over the past few years. One of the most widely used paradigms in BCI research is motor imagery (MI) [4], which involves having the subject imagine completing a task such as gripping [5] or lifting [6]. MI-BCIs are often used to assist patients with motor function issues [7,8], either for assistance with daily living or for rehabilitation training. 

According to Jeon et al. [9], MI tasks cause event-related desynchronization and synchronization in electroencephalography (EEG) [10,11]. Several feature extraction techniques have been developed to identify these activities [12,13,14]. However, because EEG is nonstationary, it is regarded as a biological signal with a low spatial resolution, high signal-to-noise ratio, and distortion [15]. Owing to these disadvantages, several studies have incorporated functional near-infrared spectroscopy (fNIRS) data to enhance the effectiveness of BCIs [16,17]. fNIRS detects the oxygen content in the blood to determine the activity level of the cerebral cortex [18]. It provides a higher spatial resolution and tolerates more motion artifacts than EEG. A 2004 study by Coyle et al. [19] used fNIRS signals to improve BCIs for the first time. The last several decades have seen a tremendous increase in the use of fNIRS in cognitive neuroscience owing to advances in brain research and cognitive neuroscience technology. 

Inadequate temporal resolution and a lack of feature extraction techniques frequently result in the subpar performance of fNIRS-based BCI systems, but EEG features can be extracted efficiently using common space patterns (CSP) [20,21]. However, the fNIRS signal is distinct from the EEG signal. EEG reflects a range of high-frequency variations and the electrophysiology of the brain. In contrast, fNIRS uses a signal that varies gradually to evaluate the effect on brain hemodynamics. Integration of the complementary qualities of EEG and fNIRS may result in a high-performance BCI because the inner edge light signal and the electrophysiological signal do not interact [22,23]. Therefore, the use of the complementary information provided by both modalities, together with the appropriate feature fusion algorithms, can lead to a higher classification accuracy compared to single-modality BCI.

According to the literature [24], two categories of EEG–fNIRS fusion frameworks exist. In the first category, known as the decision level, features of both modalities are independently entered into a classifier. For instance, Fazli et al. [17] used the band power of the EEG signal, oxyhemoglobin (ΔHbO), and deoxyhemoglobin (∆HbR) as features that were individually categorized for MI tasks. A meta-classifier refined with a feedback loop was used to integrate the results of all the classifiers. The global peak cross-validation accuracy of each classifier was used in the feedback loop to identify the optimal strategy for combining their outputs. Similarly, the spectral and temporal features of hybrid EEG–fNIRS were used to classify the auditory and visual activities [25]. Both studies reported an average accuracy improvement of 5% compared with the single modality. Shargie et al. [26] trained two support vector machine (SVM) classifiers for the two modalities, respectively, and performed a decision-level fusion. They also reported a significant improvement in the classification accuracy. 

The second category, known as feature-level fusion, entails selecting, altering, or concatenating information in the best possible manner prior to model training. Studies on the straightforward fusion of features have shown considerable improvement as compared to the results of a single modality. Shin et al. [27] fused EEG features (the log variance of the first and last three CSP components) and fNIRS features (average value of the time course and average slope) to classify MI (left-hand motor imagery (LHMI) vs. right-hand motor imagery (RHMI)) and mental arithmetic (MA) tasks (MA vs. baseline). This research group also designed a hybrid BCI model to categorize three activities (MA, RHMI, and resting-state activities) by combining features extracted from two EEG channels and two fNIRS optodes [28]. Their model correctly identified the three tasks with a 77.66 ± 12.1% accuracy. In a previous study [29], the authors investigated the performance of hybrid EEG and fNIRS signals in classifying a set of motor tasks. The study showed that hybrid EEG–fNIRS yielded a higher accuracy compared to that obtained using a single modality.

Sun et al. [30] developed a framework for feature fusion by employing linear, tensor, and p-th-order polynomials. For the MI and MA tasks, their proposed model attained classification accuracies of 77.53% and 90.19%, respectively. Their findings indicated that feature-level fusion improves the performance of hybrid BCIs. Choosing the best features via visual examination may be difficult, in particular when data from all the channels are utilized for feature extraction. According to the literature [31,32], selecting the right activity channels can help to improve the categorization ability. In addition, several studies have demonstrated the effectiveness of feature selection for BCIs [33,34,35]. Feature selection helps to reduce the dimensionality of the dataset, increase processing efficiency, and improve the classification accuracy. In a recent study, Zafar et al. [36] applied a graph convolutional network to determine the correlated activity channels in fNIRS data. A filter-based approach was employed to determine the optimal feature set for SVM training. The authors showed that their proposed method significantly improved the results compared to the full channel features. However, this methodology consists of two steps, channel selection and feature selection, making it less time-efficient. 

The use of EEG–fNIRS multimodal techniques to enhance the classification ability has yielded encouraging results. However, powerful computational strategies for the successful incorporation of these modalities are still lacking. The techniques must consider the various feature properties that must be developed in order to maximize the integration of multimodalities. Such initiatives will likely result in significant breakthroughs in packages aimed at measuring brain activity, with an increased performance compared with a single modality. The following are the main highlights of the framework presented here:

- First, the data acquired from both modalities were preprocessed to filter them and remove artifacts.
- Second, the statistical temporal features of both modalities were extracted with a 10 s interval.
- The features were fused, and the binary enhanced whale optimization algorithm (E-WOA) was used to select the optimal/efficient fused feature subset and to improve the efficiency of the multimodal characteristics by increasing their complementarity, redundancy, and significance in relation to classification labels.
- The support-vector-machine-based cost function was used to classify the data.
- An online MI dataset of 29 healthy individuals was used for validation.
- Finally, the performance results of the proposed approach were compared with those of conventional WOA, other optimization algorithms, and the published literature using the same dataset.

## 2. Proposed Framework

**Figure 1:**
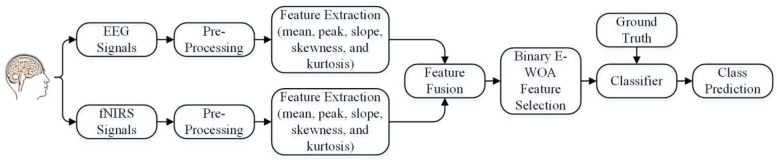
Proposed multimodality-based framework for mental task classification.

Figure 1 depicts the architecture for data acquisition, preprocessing, the extraction of temporal features, multimodal feature fusion, optimal feature selection, and model training for hybrid BCI applications. The following sections explain each step in detail.

### 2.1. Data Acquisition

**Figure 2:**
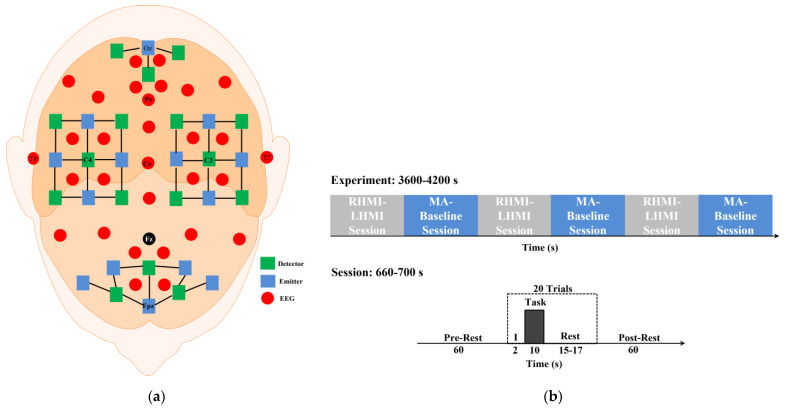
(**a**) Position of EEG and fNIRS electrodes and optodes, respectively. (**b**) Experimental paradigm.

This study used EEG and fNIRS datasets available online to validate the proposed framework [27]. The dataset contained EEG and fNIRS data from 29 healthy participants. The average age of the participants was 28.5 ± 3.7 years (14 men and 15 women). None of the participants reported having any mental, neurological, or other brain disorders. The dataset included LHMI, RHMI, MA, and baseline mental tasks. The data were collected using EEG signals from the prefrontal, motor, parietal, and occipital brain areas, utilizing Fz as a ground electrode at a sampling rate of 1000 Hz. The 10-5 international system was used for the placement of the 30 EEG electrodes. Similarly, 36 fNIRS channels were set up around Fp1, Fpz, Fp2, C3, Oz, and C4, employing 14 sources and 16 detectors at a 3 cm distance using the 10-5 international system. fNIRS data were acquired at a frequency of 2.5 Hz. The dataset consisted of triggered, fNIRS, and EEG data from 6 sessions of each of 10 trials (i.e., 30 trials per task). At the start of each session, there was a 60 s rest time, followed by 20 trials (10 per task) and another 60 s rest interval. The experiment consisted of 2 s visual instructions, a 10 s activity phase, and a randomly allotted pre-rest period of 15–17 s. Figure 2 shows the experimental paradigm and the positions of the fNIRS and EEG optodes and electrodes, respectively [27].

This study used EEG and fNIRS MI datasets to validate the proposed approach. For the MI-related activities, subjects were instructed to envision their hands opening and closing while grasping a ball. The EEG and fNIRS data were down-sampled to 200 Hz and 10 Hz, respectively, for further processing.

### 2.2. Preprocessing

In the preprocessing phase, the common average reference was used to re-reference the EEG data [37]. The EOG and EEG data were filtered between 0.5 and 5 Hz and between 0.5 and 50 Hz after re-referencing, respectively. The EOG artifacts were eliminated using a hybrid independent component analysis–regression [38]. A third-order Butterworth band-pass filter with an 8–30 Hz cutoff frequency was applied to filter the EEG data [39]. The fNIRS data were preprocessed to eliminate physiological noise such as breathing, heartbeats, and low-frequency drift. A third-order Butterworth low-pass filter with a cutoff frequency of 0.1 Hz and a Butterworth high-pass filter with a cutoff frequency of 0.01 Hz were used to preprocess the acquired fNIRS data [40,41].

### 2.3. Feature Extraction

After EEG and fNIRS data preprocessing, the features for a 10 s interval were extracted. This study focused only on the most commonly used temporal statistical features, such as the slope, peak, mean, kurtosis, and skewness [42,43,44]. The peak was defined as the highest value, and curve fitting was used to determine the slope. The following equations were used to obtain the mean (μ, Equation (1)), skewness ($S_{x}$, Equation (2)), and kurtosis ($K_{x}$, Equation (3)):

(1) $$\mu =\frac{1}{N}\sum_{k=k_{1}}^{k_{2}}Y(k)$$

(2) $$S_{x}=\frac{E_{x}{\left(Y_{x}-\mu_{x}\right)}^{3}}{\sigma^{3}}$$

(3) $$K_{x}=\frac{E_{x}{\left(Y_{x}-\mu_{x}\right)}^{4}}{\sigma^{4}}$$

where Y corresponds to the input signal (EEG and fNIRS), σ to the standard deviation, and $E_{x}$ to the statistical expectation. 

The features were extracted using all EEG and fNIRS channels. In total, 180 features (36 channels × 5 features) were extracted from the fNIRS data, whereas the EEG data contained 150 features (30 channels × 5 features). Subsequently, all features were normalized separately for both modalities. Finally, all the features from both modalities were concatenated (f_fNIRS_:f_EEG_). The final fused feature vector comprised 330 features (180 (f_fNIRS_) + 150 (f_EEG_)). An optimal feature selection approach is required to obtain the most reliable information for a robust and highly accurate EEG-fNRIS-based BCI. 

### 2.4. Optimal Feature Selection Approach

Feature selection is a crucial step in EEG–fNIRS-based BCI systems. External devices can be controlled and environmental communication can be performed through EEG and fNIRS by measuring the electrical or hemodynamic activity of the brain. Owing to the complexity, high dimensionality, noise, and abundance of redundant and irrelevant information present in EEG and fNIRS data, feature selection is crucial. The accuracy and speed of a BCI system can be improved using feature selection, which also helps to reduce computational complexity and improve the interpretability of the results.

Filter and wrapper methods are the two primary divisions of feature selection approaches. Filter methods rank features according to their relevance and redundancy using statistical or information-theoretic metrics and then select the top-ranked features for further analysis. Wrapper approaches analyze the performance of a BCI system using several feature subsets and select the feature subset that provides the best performance using machine learning algorithms. In this study, a binary E-WOA wrapper-based feature selection method was applied to enhance the efficiency of the hybrid BCI.

#### 2.4.1. Whale Optimization Algorithm (WOA)

The WOA is a metaheuristic optimization technique that was introduced in 2016 by Mirjalili [45]. It is based on the hunting behavior of humpback whales and is intended to address various optimization challenges. The bubble net feeding technique employed by humpback whales, whereby they ascend near to the surface and swim in a spiral pattern to catch their prey in a net of bubbles, serves as the model for the WOA [45]. The WOA keeps track of a population of potential solutions, or “whales”. The algorithm updates the whales’ location using three forms of movement: encircling prey, spiral bubble net attack, and searching for prey. The encircling of prey refers to the act of traveling in a circle around the optimal solution so as to converge on it. Spiral bubble net attack entails traveling toward the best solution available in the given moment, and searching for prey involves random movement.

Assuming that $X(t)=\left(x_{1}(t),x_{1}(t),\ldots x_{m}(t)\right)$ represents the population of whales in an *N*-dimensional search space, the position of each whale is represented by $X_{i}(t)=\left(x_{i,1}(t),x_{i,1}(t),\ldots x_{i,D}(t)\right)$. The matrix X(1) is randomly initialized for the first and subsequent iterations in the problem space. The vector X(t) values update themselves using the three previously defined WOA modeling methods. During optimization, WOA considers the rate of probability (ρ) that each $X_{i}(t)$ will alternate between encircling/searching for prey and spiral bubble net attacking strategies using the coefficient vector ($A_{i}(t)$). The updated position of the whale can be computed using Equation (4):

(4) $$X_{i}(t)=\{\begin{array}{c} \mathrm{Encircling}\;\mathrm{prey}\;\left(\rho_{i}(t)<0.5\right)\;\mathrm{and}\;\left(\left|A_{i}(t)\right|<1\right)\\ \mathrm{Search}\;\mathrm{for}\;\mathrm{prey}\;\left(\rho_{i}(t)<0.5\right)\;\mathrm{and}\;\left(\left|A_{i}(t)\right|\geq 1\right)\\ \mathrm{Spiral}\;\mathrm{bubble}-\mathrm{net}\;\mathrm{attacking}\;\left(\rho_{i}(t)\geq 0.5\right) \end{array},0<\rho_{i}(t)<1$$

(5) $$A_{i}(t)=2\times a_{i}(t)\times rand-a_{i}(t)$$

where $\;a_{i}(t)$ represents the linearly decreasing variable and can be computed using Equation (6):

(6) $$a_{i}(t)=2-t\times \left(\frac{2}{MaxIt}\right)$$

The mathematical model of the method of encircling prey is presented in the following equations:

(7) $$\begin{array}{c} \;\;\;\;\;\;\;\;\;\;\;\;\;\;\;\;\;X_{i}(t+1)=X_{best}(t)-A_{i}(t)\times D(t)\\ \;\;\;\;\;\;\;\;\;\;\;\;\;\;\;\;\;\;\;\;\;\;\;\;\;\;\;\;\;D(t)=\left|C_{i}(t)\times X_{best}(t)-X_{i}(t)\right|\\ C_{i}(t)=2\times rand \end{array}\}$$

where D(t) represents the distance between the current and optimal positions of the whale, and $C_{i}(t)$ represents the coefficient vector at the t iteration. The search for prey can be modeled as follows:

(8) $$\begin{array}{c} X_{i}(t+1)=X_{rnd}(t)-A_{i}(t)\times D(t)\\ \;\;\;\;\;\;\;\;\;\;\;\;D(t)=\left|C_{i}(t)\times X_{rnd}(t)-X_{i}(t)\right| \end{array}\}$$

The spiral bubble net attacking method can be modeled in turn using Equation (9), where C represents the logarithmic spiral shape:

(9) $$\begin{array}{c} \;\;\;\;\;\;\;\;\;\;\;\;\;\;\;\;\;\;\;\;\;\;\;X_{i}(t+1)=D^{\prime }(t)\times \exp^{cl}\times \cos(2\pi l)+X_{best}(t)\\ D^{\prime }(t)=\left|X_{best}(t)-X_{i}(t)\right| \end{array}\},-1\leq l\leq 1$$

Although the WOA is a well-known optimization method, it is still affected by early convergence, limited population diversity, and the misalignment of search strategies [46]. Therefore, Shahraki et al. [47] proposed enhanced WOA (E-WOA) and binary E-WOA variants for feature selection. 

#### 2.4.2. Enhanced WOA (E-WOA)

In E-WOA, a pooling technique and three effective search tactics—migration, preferential selection, and enriched surrounding prey—enhance the effectiveness of conventional WOAs [47]. The pooling mechanism maintains population diversity by fusing unsuccessful solutions from each iteration with a successful solution. Advanced search techniques are also included to strengthen the conventional WOA search techniques.

The E-WOA pool matrix (P(1),P(2),…,P(k)) with the members $P_{i}=P_{i}(1),P_{i}(2),\ldots ,P_{i}(k)$ is computed using Equation (10) in the pooling mechanism at each iteration:

(10) $$P_{i}(t)=B_{i}(t)\times X_{brnd}(t)\times {\bar{B}}_{i}(t)+X_{worst}(t)$$

where $X_{brnd}(t)$ represents a random position near the best whale $X_{best}(t)$, and $X_{worst}(t)$ represents the worst whale at a given iteration. $B_{i}(t)$ and ${\bar{B}}_{i}(t)$ are the random and reverse vectors, meaning that the corresponding values of zero-valued elements in ${\bar{B}}_{i}(t)$ have a value of one and the corresponding values of non-zero-valued elements in $B_{i}(t)$ have a value of zero in ${\bar{B}}_{i}(t)$. The pooling technique uses a crossover operator to improve the diversity and combines the least viable solution with a promising solution. A new solution is updated using the existing pool member.

The migrating search technique randomly separates a proportion of the whales from the group using Equation (11) to cover previously unexplored areas and enhance exploration. Moreover, it is anticipated that the separated whales will boost population variety, which will help to reduce local optima trapping:

(11) $$\begin{array}{c} \;X_{i}(t+1)=X_{rnd}(t)-X_{brnd}(t)\\ \;\;\;\;X_{rnd}(t)=rand\times \left(\delta_{\max}-\delta_{\min}\right)+\delta_{\min}\\ \;\;\;X_{brnd}(t)=rand\left(\delta_{best\_\max}-\delta_{best\_\min}\right)+\delta_{best\_\min} \end{array}\}$$

where $\delta_{best\_\max}$ and $\delta_{best\_\min}$ represent the upper and lower boundaries of $X_{best}(t)$.

The preferential selection strategy enhances the search for prey approach. The preferential selection approach is modeled using Equation (12):

(12) $$X_{i}(t+1)=X_{i}(t)+A_{i}(t)\times \left(C_{i}(t)\times P_{rnd1}(t)-P_{rnd2}(t)\right)$$

where $P_{rnd1}(t)$ and $P_{rnd2}(t)$ are randomly selected using (P(1),P(2),…,P(k)). 

The encircling prey method from the conventional WOA is updated using the following equation:

(13) $$\begin{array}{c} X_{i}(t+1)=X_{best}(t)-A_{i}(t)\times D^{\prime }(t)\\ \;\;\;\;\;\;\;\;\;\;\;\;\;\;D^{\prime }(t)=\left|C_{i}(t)\times X_{best}(t)-P_{rnd3}(t)\right| \end{array}\}$$

**Figure 3:**
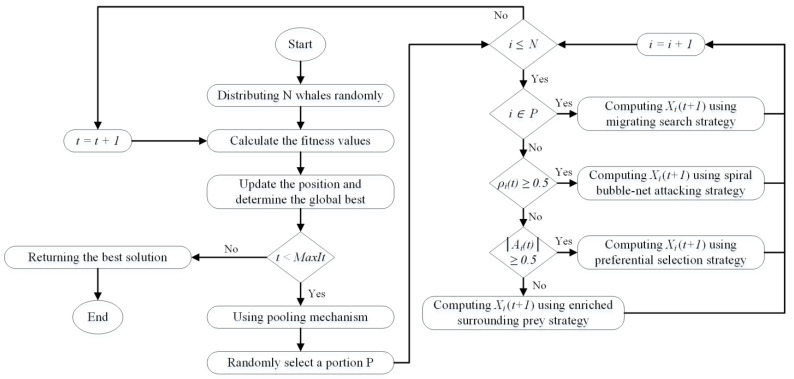
Flowchart of E-WOA.

Using a matrix pool, $P_{rnd3}(t)$ can be randomly selected. The flowchart corresponding to E-WOA is shown in Figure 3.

**Figure 4:**
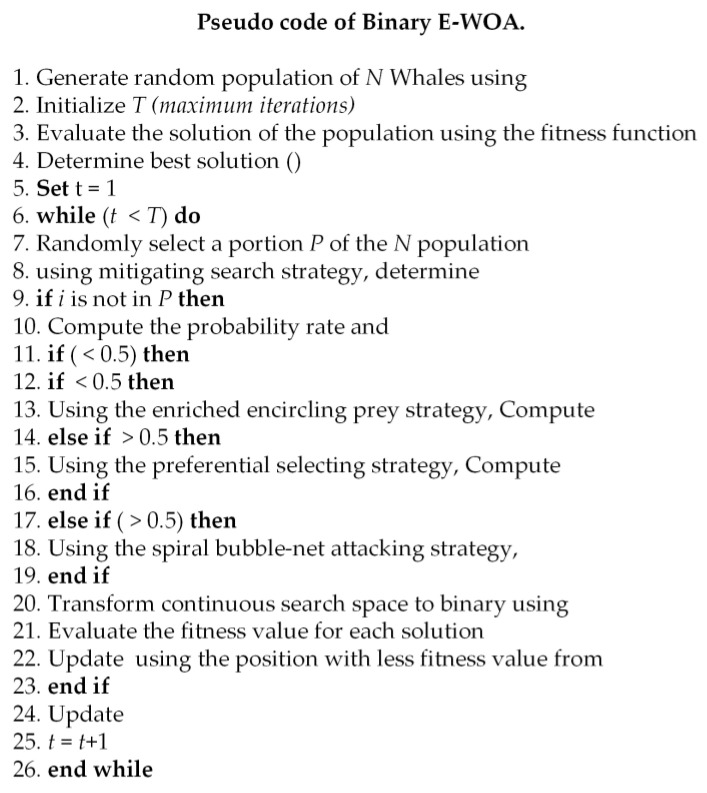
Pseudo-code of binary E-WOA for feature selection [47].

Shahraki et al. [47] also proposed the binary E-WOA for feature selection. The most significant/optimal features or variables associated with certain medical conditions can be successfully identified using binary optimization feature selection algorithms, making them useful for medical applications. Binary optimization feature selection algorithms assist in increasing the accuracy and efficacy of medical diagnoses and treatments by limiting the number of features or variables to those that are most important. The pseudo-code for the binary E-WOA is shown in Figure 4. 

### 2.5. Support-Vector-Machine-Based Classification

The supervised classifier SVM has been used in several studies [42,48,49]. The main goal of SVM is to identify the optimal discriminative hyperplane that maximizes the margin between two classes. The so-called support vectors, or vectors on the margin, can be utilized to describe the ideal hyperplane. The hyperplane for a 2D feature space can be defined as follows:

(14) f(x)=w⋅x+b

where *x* denotes the input test data, *w* denotes the weight vector, and *b* denotes the bias factor. Projecting the test data from the input onto *w*, one can forecast the corresponding class output (i.e., response variable). 

## 3. Results 

As previously mentioned, this study used an open dataset of MI brain tasks to validate the proposed approach. Each trial was 10 s long and consisted of 30 trials for each task and each participant (30 in total). The performance of the temporal features of the unimodal data (EEG and ΔHbO-fNIRS) for all the channels using a 10 s interval is presented in Table 1. In total, 80% of the data were used for training, whereas the remaining 20% were reserved for the testing of the developed model. The number of features and the classification accuracy were used as comparison metrics. Equation (15) was used to compute the classification accuracy:

(15) $$\mathrm{Classification}\;\mathrm{Accuracy}\;(\%)=\frac{\mathrm{Correctly}\;\mathrm{classified}\;\mathrm{trail}}{\mathrm{Total}\;\mathrm{no}.\;\mathrm{of}\;\mathrm{trails}}\times 100$$

After a critical evaluation of the findings, it was revealed that the EEG and fNIRS data showed the highest classification accuracies (68.33% and 82.5%) for Subjects 26 and 28, respectively. The average accuracies of EEG and fNIRS were 53.59 ± 7.88 and 58.33 ± 10.13, respectively. The average accuracies of the two modalities were very low. Therefore, we applied the conventional WOA and binary E-WOA approaches to check the performance of the fused dataset. The fused feature vector contained 330 features (150 (EEG) + 180 (fNIRS)). The population selected for both optimizations was 10, with a maximum of 50 iterations. The results of the 10 runs are reported in Table 2.

**Figure 5:**
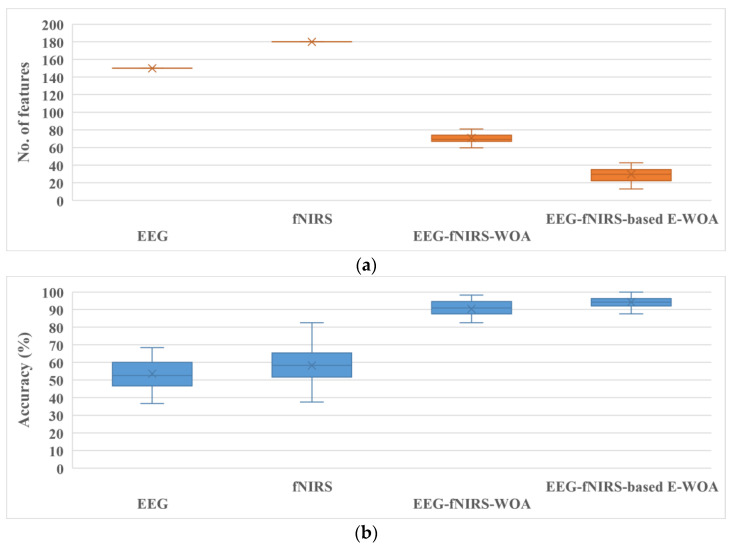
Performance of a single modality and hybrid modalities with conventional WOA and binary E-WOA (**a**) in terms of the number of features and (**b**) in terms of accuracy.

As shown in Table 2, both optimization algorithms significantly enhanced the classification performance of the hybrid features from both modalities. Both wrapper-based optimization algorithms tested the effectiveness of the features by training a model with a subset of features. It is evident from Table 2 that the conventional WOA and binary E-WOA approaches enhanced the classification accuracy of Subject 1 using only 97 and 55 features, respectively. The binary E-WOA increased the classification accuracy by almost 9% compared to the conventional WOA. The classification accuracy of Subject 28 was 99.17 ± 2.64, nearly 16.5% higher than that obtained with fNIRS. Subject 28 used only 26.6 ± 13.06 optimal features from both modalities out of the total number of features (330). The box plot shown in Figure 5 summarizes the results in a simple manner.

**Figure 6:**
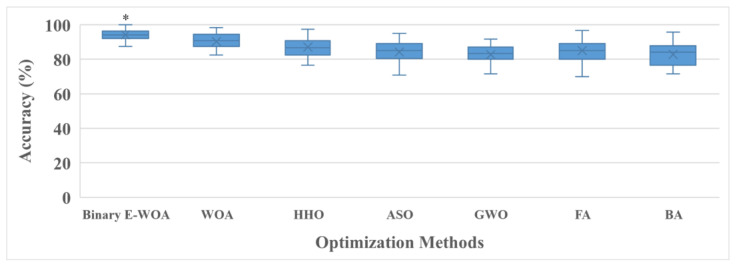
Comparison of various wrapper-based approaches. HHO: Harris hawk optimization [50], ASO: atom search optimization [51]; GWO: grey wolf optimization [52], FA: firefly algorithm [53], and BA: bat algorithm [54]. * *p* < 0.01.

As shown in Figure 5, the hybrid modalities outperformed the solo modalities using the conventional WOA and binary E-WOA. The conventional WOA and binary E-WOA demonstrated a high classification accuracy (90.37 ± 7.66% and 94.22 ± 5.39%, respectively). The proposed approach (binary E-WOA) only required 29.62 ± 20.78 features to train the model. In contrast, the conventional WOA required 70.81 ± 11.63 features, as depicted in Figure 5. Furthermore, the proposed approach (EEG–fNIRS-based binary E-WOA) was compared to other wrapper-based techniques [50,51,52,53,54,55]. These findings are shown in Figure 6.

The comparison of the results revealed that the EEG–fNIRS-based binary E-WOA outperformed all the other optimization algorithms in terms of the classification rate. A two-sample t-test also proved the reliability and adaptability of the results, as shown in Figure 6. Compared with the outcomes of the other optimization techniques, the binary E-WOA findings were highly accurate (*p* < 0.01). The proposed approach was also compared with those previously described in the literature, and the results are listed in Table 3.

## 4. Discussion

Multimodal hybrid techniques outperform unimodal data in terms of classification performance using conventional machine learning or deep learning techniques [59]. Although there is variability between EEG and fNIRS data, it is not as significant as initially believed based on the signal sources. Moreover, the adoption of certain effective techniques for multimodal fusion improves BCI performance. Owing to the different acquisition methods, noise sensitivities, and temporal and spatial resolutions, information from several modalities is not directly compatible. One of the main challenges in the development of hybrid BCIs is finding methods to integrate complementary information, maximize its advantages, and overcome the limitations of a single model.

Shin et al. [27] computed common spatial patterns for EEG and two statistical features for fNIRS and utilized linear discriminant analysis for MI task classification. The reported accuracy for the hybrid BCI was only 67.5%. Similarly, in another study [56], the authors developed an independent decision path fusion approach. He et al. [58] developed a neural network for a hybrid BCI model. However, the computational complexity of the model was high. 

In this study, the statistical features of the two modalities were merged. Wrapper-based and binary E-WOAs were applied to select the optimal features. The results show that the binary E-WOA outperformed the conventional WOA in terms of accuracy (Table 2 and Figure 5). The ability of the binary E-WOA to detect and select more appropriate features from the hybrid EEG and fNIRS data for the classification task can be attributed to its effective search strategy. Feature selection in the conventional WOA is entirely determined by the objective function, which cannot accurately reflect the complexity and variety of brain MI datasets. This may lead to the selection of irrelevant or less-than-ideal features, which may, in turn, lead to subpar classification results, as shown in Figure 5. On the other hand, the binary E-WOA utilizes a more complex feature selection technique that incorporates the objective function and a collection of three advanced search strategies to assist in the search for optimal features, as discussed in Section 2.4.2. The three effective search strategies allow the binary E-WOA to explore the feature set more effectively and discover a more diverse collection of optimal features. This results in an improved classification accuracy and a more robust classification model. Moreover, when the results of the proposed approach were compared with those of other feature selection approaches, the binary E-WOA outperformed them and provided more robust and accurate classification results, further validating the reliability of the proposed framework. Therefore, it can be concluded that the proposed binary E-WOA hybrid EEG–fNIRS framework can be used for BCI applications. 

## 5. Conclusions

In this study, an enhanced binary variant of the WOA was applied to improve the performance of an EEG–fNIRS-based BCI. The binary E-WOA was used to select the optimal statistical features for both modalities. The three novel search strategies enhanced the classification performance of the conventional WOA. After extensive training and testing, the proposed framework classified the MI task with a 94.22 ± 5.39% accuracy using features from both modalities. The results also suggested that the improved binary variant increased the classification accuracy for MI tasks by more than 3.85% compared to the conventional WOA. Furthermore, the results were compared with those obtained using other metaheuristic algorithms, validating the high performance of the proposed framework for classification. To demonstrate the statistical significance of the results, a two-sample t-test was performed, which revealed that the differences in accuracy, as compared with all the other approaches, were significant (*p* < 0.01). Therefore, the framework presented here may be useful for hybrid EEG–fNIRS BCI applications. In summary, the present study indicates that the binary E-WOA hybrid EEG–fNIRS could be used as a potential tool for various applications, including brain-controlled devices, communication, and neurorehabilitation.

## Figures and Tables

**Table 1:** Performance of EEG and fNIRS for LHMI vs. RHMI.

Subject	EEG		fNIRS	
	No. of Features	Accuracy (%)	No. of Features	Accuracy (%)
1	150	45.83 ± 11.95	180	50 ± 12.42
2		51.67 ± 12.91		66.67 ± 14.16
3		52.5 ± 11.82		71.67 ± 18.92
4		63.33 ± 12.55		62.5 ± 9
5		57.5 ± 10.72		46.67 ± 9.78
6		52.5 ± 6.86		60 ± 12.91
7		50.83 ± 14.41		51.67 ± 10.97
8		45 ± 11.92		58.33 ± 18.43
9		61.67 ± 10.54		53.33 ± 17.66
10		42.5 ± 12.08		65.83 ± 10.72
11		55.83 ± 12.45		55 ± 11.92
12		47.5 ± 11.82		58.33 ± 11.11
13		51.67 ± 12.91		65 ± 15.61
14		55 ± 18.51		53.33 ± 14.27
15		45.83 ± 7.08		51.67 ± 16.57
16		58.33 ± 15.71		54.17 ± 9
17		42.5 ± 12.08		44.17 ± 11.15
18		51.67 ± 14.59		64.17 ± 10.43
19		57.5 ± 10.72		37.5 ± 10.58
20		61.67 ± 13.72		65 ± 15.61
21		51.67 ± 12.3		65.83 ± 12.7
22		36.67 ± 5.83		38.33 ± 9.78
23		64.17 ± 14.72		51.67 ± 10.24
24		50.83 ± 13.29		55.83 ± 4.03
25		66.67 ± 14.16		60.83 ± 11.15
26		68.33 ± 6.57		66.67 ± 14.7
27		63.33 ± 5.83		74.17 ± 17.32
28		55.83 ± 9.66		82.5 ± 7.3
29		45.83 ± 15.34		60.83 ± 13.64
Average		53.59 ± 7.88		58.33 ± 10.13

**Table 2:** Performance of the conventional WOA and binary E-WOA for the hybrid (EEG–fNIRS) dataset.

Subject	Conventional WOA		Binary E-WOA	
	No. of Features	Accuracy (%)	No. of Features	Accuracy (%)
1	96.8 ± 28.11	82.5 ± 13.86	54.4 ± 23.89	91.67 ± 5.56
2	69.3 ± 10.33	95 ± 4.3	22.5 ± 26.44	97.5 ± 7.91
3	81 ± 31.06	90.83 ± 10.72	37.5 ± 19.45	96.67 ± 4.3
4	77.9 ± 12.12	87.5 ± 9.82	35.6 ± 34.7	90 ± 6.57
5	74.2 ± 12.55	82.5 ± 9.17	33.6 ± 21.11	94.17 ± 5.62
6	63.3 ± 9.65	85.83 ± 11.82	12.9 ± 9.24	92.5 ± 4.73
7	67.1 ± 10.24	88.33 ± 5.83	23.1 ± 23.48	93.33 ± 3.51
8	77.5 ± 14.97	93.33 ± 8.61	31.7 ± 15.38	96.67 ± 4.3
9	74 ± 12.44	94.17 ± 5.62	58.2 ± 38.31	95.83 ± 4.39
10	73 ± 8.62	90 ± 7.66	42.6 ± 22.78	92.5 ± 8.29
11	73.3 ± 9.07	89.17 ± 7.91	18.8 ± 21.09	92.5 ± 4.73
12	61.4 ± 10.5	82.5 ± 7.3	13.2 ± 9.53	90 ± 7.66
13	74.7 ± 10.86	95.83 ± 5.89	29.8 ± 23.38	95 ± 5.83
14	59.7 ± 8.26	92.5 ± 6.15	22.1 ± 17.49	95.83 ± 5.89
15	68 ± 6.41	87.5 ± 8.1	21.9 ± 14.9	90.83 ± 7.3
16	72.3 ± 10.79	89.17 ± 7.91	30.7 ± 24.91	91.67 ± 6.8
17	75.5 ± 10.62	95 ± 5.83	29 ± 21.29	94.17 ± 5.62
18	65.1 ± 6.3	92.5 ± 4.73	36.2 ± 15.5	95.83 ± 5.89
19	69.1 ± 10.29	93.33 ± 6.57	30.4 ± 26.88	96.67 ± 4.3
20	69.3 ± 10.88	94.17 ± 5.62	35.8 ± 15.45	95 ± 5.83
21	66.9 ± 8.54	90.83 ± 6.15	28.9 ± 17.07	94.17 ± 6.86
22	66.1 ± 12.72	76.67 ± 13.49	22.2 ± 25.66	87.5 ± 8.1
23	68.7 ± 11.21	95 ± 8.05	34.2 ± 36.01	95 ± 5.83
24	73.4 ± 10.38	88.33 ± 8.96	32.2 ± 17.86	89.17 ± 6.86
25	68.2 ± 14.31	92.5 ± 9.98	30.9 ± 27.94	95.83 ± 5.89
26	69.5 ± 8.51	96.67 ± 5.83	27.3 ± 17.55	100 ± 0
27	59.9 ± 8.81	97.5 ± 4.03	22.5 ± 9.57	100 ± 0
28	70.5 ± 10.32	98.33 ± 3.51	26.6 ± 13.06	99.17 ± 2.64
29	68 ± 8.6	83.33 ± 8.78	14.3 ± 12.79	93.33 ± 5.27
Average		90.37 ± 7.66		94.22 ± 5.39

**Table 3:** Accuracy comparison with various studies.

Reference	Year	Approach	Accuracy (%)
Shin et al. [27]	2016	Common spatial pattern, mean, slope, shrinkage LDA	67.5
Sun et al. [30]	2020	p-th-Order polynomial fusion	77.53
Jiang et al. [56]	2019	Independent decision path fusion	78.56
Wang et al. [57]	2022	R-CSP-E transfer and ensemble learning	66.83
He et al. [58]	2022	End-to-end multimodal multitask neural network	82.11
Present Study	2023	Temporal features and binary E-WOA	94.22 ± 5.39

## Data Availability

The data used to support the findings of this study are included in the article.

## References

[1] Nicolas-Alonso L.F., Gomez-Gil J. (2012). Brain computer interfaces, a review. Sensors.

[2] Khosrowabadi R., Quek C., Ang K.K., Tung S.W., Heijnen M. A Brain-Computer Interface for classifying EEG correlates of chronic mental stress. Proceedings of the 2011 International Joint Conference on Neural Networks.

[3] Hekmatmanesh A., Nardelli P.H., Handroos H. (2021). Review of the state-of-the-art of brain-controlled vehicles. IEEE Access.

[4] Kaiser V., Kreilinger A., Müller-Putz G.R., Neuper C. (2011). First steps toward a motor imagery based stroke BCI: New strategy to set up a classifier. Front. Neurosci..

[5] Herath H.M.K.K.M.B., de Mel W.R. (2021). Controlling an Anatomical Robot Hand Using the Brain-Computer Interface Based on Motor Imagery. Adv. Hum.-Comput. Interact..

[6] Kasemsumran P., Boonchieng E. (2019). EEG-based motor imagery classification using novel adaptive threshold feature extraction and string grammar fuzzy K-nearest neighbor classification. J. Comput..

[7] Ang K.K., Guan C.T., Chua K.S.G., Ang B.T., Kuah C., Wang C.C., Phua K.S., Chin Z.Y., Zhang H.H., IEEE Clinical study of neurorehabilitation in stroke using EEG-based motor imagery brain-computer interface with robotic feedback. Proceedings of the 2010 Annual International Conference of the IEEE Engineering in Medicine and Biology Society (EMBC).

[8] Lule D., Diekmann V., Kassubek J., Kurt A., Birbaumer N., Ludolph A.C., Kraft E. (2007). Cortical plasticity in amyotrophic lateral sclerosis: Motor imagery and function. Neurorehabilit. Neural Repair.

[9] Jeon Y., Nam C.S., Kim Y.J., Whang M.C. (2011). Event-related (De)synchronization (ERD/ERS) during motor imagery tasks: Implications for brain-computer interfaces. Int. J. Ind. Ergon..

[10] Hekmatmanesh A., Wu H., Motie-Nasrabadi A., Li M., Handroos H. (2019). Combination of discrete wavelet packet transform with detrended fluctuation analysis using customized mother wavelet with the aim of an imagery-motor control interface for an exoskeleton. Multimed. Tools Appl..

[11] Hekmatmanesh A., Asl R.M., Wu H., Handroos H. (2019). EEG control of a bionic hand with imagination based on chaotic approximation of largest Lyapunov exponent: A single trial BCI application study. IEEE Access.

[12] Dagdevir E., Tokmakci M. (2021). Optimization of preprocessing stage in EEG based BCI systems in terms of accuracy and timing cost. Biomed. Signal Process. Control..

[13] Sadiq M.T., Yu X.J., Yuan Z.H., Fan Z.M., Rehman A.U., Ullah I., Li G.Q., Xiao G.X. (2019). Motor Imagery EEG Signals Decoding by Multivariate Empirical Wavelet Transform-Based Framework for Robust Brain-Computer Interfaces. IEEE Access.

[14] Hekmatmanesh A., Wu H., Jamaloo F., Li M., Handroos H. (2020). A combination of CSP-based method with soft margin SVM classifier and generalized RBF kernel for imagery-based brain computer interface applications. Multimed. Tools Appl..

[15] Hallez H., Vanrumste B., Grech R., Muscat J., De Clercq W., Vergult A., D’Asseler Y., Camilleri K.P., Fabri S.G., Van Huffel S. (2007). Review on solving the forward problem in EEG source analysis. J. Neuroeng. Rehabil..

[16] Quaresima V., Ferrari M. (2019). A Mini-Review on Functional Near-Infrared Spectroscopy (fNIRS): Where Do We Stand, and Where Should We Go?. Photonics.

[17] Fazli S., Mehnert J., Steinbrink J., Curio G., Villringer A., Muller K.R., Blankertz B. (2012). Enhanced performance by a hybrid NIRS-EEG brain computer interface. Neuroimage.

[18] Ferrari M., Quaresima V. (2012). A brief review on the history of human functional near-infrared spectroscopy (fNIRS) development and fields of application. Neuroimage.

[19] Coyle S., Ward T., Markham C., McDarby G. (2004). On the suitability of near-infrared (NIR) systems for next-generation brain–computer interfaces. Physiol. Meas..

[20] Spüler M., Walter A., Rosenstiel W., Bogdan M. (2013). Spatial filtering based on canonical correlation analysis for classification of evoked or event-related potentials in EEG data. IEEE Trans. Neural Syst. Rehabil. Eng..

[21] Wu D., King J.-T., Chuang C.-H., Lin C.-T., Jung T.-P. (2017). Spatial filtering for EEG-based regression problems in brain–computer interface (BCI). IEEE Trans. Fuzzy Syst..

[22] Pfurtscheller G., Allison B.Z., Bauernfeind G., Brunner C., Solis Escalante T., Scherer R., Zander T.O., Mueller-Putz G., Neuper C., Birbaumer N. (2010). The hybrid BCI. Front. Neurosci..

[23] Dähne S., Biessmann F., Samek W., Haufe S., Goltz D., Gundlach C., Villringer A., Fazli S., Müller K.-R. (2015). Multivariate machine learning methods for fusing multimodal functional neuroimaging data. Proc. IEEE.

[24] Deligani R.J., Borgheai S.B., McLinden J., Shahriari Y. (2021). Multimodal fusion of EEG-fNIRS: A mutual information-based hybrid classification framework. Biomed. Opt. Express.

[25] Putze F., Hesslinger S., Tse C.-Y., Huang Y., Herff C., Guan C., Schultz T. (2014). Hybrid fNIRS-EEG based classification of auditory and visual perception processes. Front. Neurosci..

[26] Al-Shargie F., Tang T.B., Kiguchi M. (2017). Stress Assessment Based on Decision Fusion of EEG and fNIRS Signals. IEEE Access.

[27] Shin J., von Lühmann A., Blankertz B., Kim D.-W., Jeong J., Hwang H.-J., Müller K.-R. (2016). Open access dataset for EEG+ NIRS single-trial classification. IEEE Trans. Neural Syst. Rehabil. Eng..

[28] Kwon J., Shin J., Im C.-H. (2020). Toward a compact hybrid brain-computer interface (BCI): Performance evaluation of multi-class hybrid EEG-fNIRS BCIs with limited number of channels. PLoS ONE.

[29] Buccino A.P., Keles H.O., Omurtag A. (2016). Hybrid EEG-fNIRS asynchronous brain-computer interface for multiple motor tasks. PLoS ONE.

[30] Sun Z., Huang Z., Duan F., Liu Y. (2020). A Novel Multimodal Approach for Hybrid Brain–Computer Interface. IEEE Access.

[31] Zafar A., Ghafoor U., Yaqub M.A., Hong K.-S. (2019). Initial-dip-based classification for fNIRS-BCI. Proceedings of the Neural Imaging and Sensing 2019.

[32] Asam M., Khan S.H., Akbar A., Bibi S., Jamal T., Khan A., Ghafoor U., Bhutta M.R. (2022). IoT malware detection architecture using a novel channel boosted and squeezed CNN. Sci. Rep..

[33] Qi Y., Ding F., Xu F., Yang J. (2020). Channel and Feature Selection for a Motor Imagery-Based BCI System Using Multilevel Particle Swarm Optimization. Comput. Intell. Neurosci..

[34] Naseer N., Hong K.-S. (2015). fNIRS-based brain-computer interfaces: A review. Front. Hum. Neurosci..

[35] Nazeer H., Naseer N., Khan R.A., Noori F.M., Qureshi N.K., Khan U.S., Khan M.J. (2020). Enhancing classification accuracy of fNIRS-BCI using features acquired from vector-based phase analysis. J. Neural Eng..

[36] Zafar A., Dad Kallu K., Atif Yaqub M., Ali M.U., Hyuk Byun J., Yoon M., Su Kim K. (2023). A Hybrid GCN and Filter-Based Framework for Channel and Feature Selection: An fNIRS-BCI Study. Int. J. Intell. Syst..

[37] Dien J. (1998). Issues in the application of the average reference: Review, critiques, and recommendations. Behav. Res. Methods Instrum. Comput..

[38] Mannan M.M.N., Jeong M.Y., Kamran M.A. (2016). Hybrid ICA—Regression: Automatic identification and removal of ocular artifacts from electroencephalographic signals. Front. Hum. Neurosci..

[39] Suk H.I., Lee S.W. (2011). Subject and class specific frequency bands selection for multiclass motor imagery classification. Int. J. Imaging Syst. Technol..

[40] Zafar A., Hong K.-S. (2018). Neuronal activation detection using vector phase analysis with dual threshold circles: A functional near-infrared spectroscopy study. Int. J. Neural Syst..

[41] Zafar A., Hong K.-S. (2020). Reduction of onset delay in functional near-infrared spectroscopy: Prediction of HbO/HbR signals. Front. Neurorobot..

[42] Hong K.-S., Khan M.J., Hong M.J. (2018). Feature extraction and classification methods for hybrid fNIRS-EEG brain-computer interfaces. Front. Hum. Neurosci..

[43] Hasan M.A.H., Khan M.U., Mishra D. (2020). A Computationally Efficient Method for Hybrid EEG-fNIRS BCI Based on the Pearson Correlation. BioMed Res. Int..

[44] Zafar A., Hussain S.J., Ali M.U., Lee S.W. (2023). Metaheuristic Optimization-Based Feature Selection for Imagery and Arithmetic Tasks: An fNIRS Study. Sensors.

[45] Mirjalili S., Lewis A. (2016). The Whale Optimization Algorithm. Adv. Eng. Softw..

[46] Gharehchopogh F.S., Gholizadeh H. (2019). A comprehensive survey: Whale Optimization Algorithm and its applications. Swarm Evol. Comput..

[47] Nadimi-Shahraki M.H., Zamani H., Mirjalili S. (2022). Enhanced whale optimization algorithm for medical feature selection: A COVID-19 case study. Comput. Biol. Med..

[48] Aydin E.A. (2020). Subject-Specific feature selection for near infrared spectroscopy based brain-computer interfaces. Comput. Methods Programs Biomed..

[49] Hong K.-S., Zafar A. (2018). Existence of initial dip for BCI: An illusion or reality. Front. Neurorobot..

[50] Bairathi D., Gopalani D. A novel swarm intelligence based optimization method: Harris’ hawk optimization. Proceedings of the Intelligent Systems Design and Applications: 18th International Conference on Intelligent Systems Design and Applications (ISDA 2018).

[51] Kamel S., Hamour H., Ahmed M.H., Nasrat L. Atom Search optimization Algorithm for Optimal Radial Distribution System Reconfiguration. Proceedings of the 2019 International Conference on Computer, Control, Electrical, and Electronics Engineering (ICCCEEE).

[52] Mirjalili S., Mirjalili S.M., Lewis A. (2014). Grey wolf optimizer. Adv. Eng. Softw..

[53] Fister I., Fister Jr I., Yang X.-S., Brest J. (2013). A comprehensive review of firefly algorithms. Swarm Evol. Comput..

[54] Yang X.-S. (2021). Chapter 11—Bat Algorithms. Nature-Inspired Optimization Algorithms.

[55] Ali M.U., Hussain S.J., Zafar A., Bhutta M.R., Lee S.W. (2023). WBM-DLNets: Wrapper-Based Metaheuristic Deep Learning Networks Feature Optimization for Enhancing Brain Tumor Detection. Bioengineering.

[56] Jiang X., Gu X., Xu K., Ren H., Chen W. (2019). Independent decision path fusion for bimodal asynchronous brain–computer interface to discriminate multiclass mental states. IEEE Access.

[57] Wang Y., Yang Z., Ji H., Li J., Liu L., Zhuang J. (2022). Cross-Modal Transfer Learning from EEG to Functional Near-Infrared Spectroscopy for Classification Task in Brain-Computer Interface System. Front. Psychol..

[58] He Q., Feng L., Jiang G., Xie P. (2022). Multimodal Multitask Neural Network for Motor Imagery Classification With EEG and fNIRS Signals. IEEE Sens. J..

[59] Li Y., Zhang X., Ming D. (2023). Early-stage fusion of EEG and fNIRS improves classification of motor imagery. Front. Neurosci..

